# E-health literacy in older adults: an evolutionary concept analysis

**DOI:** 10.1186/s12911-022-01761-5

**Published:** 2022-01-31

**Authors:** Sun Ok Jung, Yoon Hee Son, Eunju Choi

**Affiliations:** College of Nursing, Ewha Womans University, 52 Ewhayeodae-gil, Seodaemun-gu, Seoul, 03760 Republic of Korea

**Keywords:** Aging, Concept analysis, E-health, Health literacy

## Abstract

**Background:**

Internet technologies have become important for older adults to not only seek, understand, and evaluate information on health management but also apply and share acquired knowledge. Despite the disparity in e-health literacy among older adults, which affects health outcomes, its conceptual definition has not been distinctly clarified in previous studies. This study aimed to analyse the concept of e-health literacy among older adults and to identify its contexts in the nursing field.

**Methods:**

We identified concepts, attributes, antecedents, and consequences of e-health literacy in older adults using Rodgers’ evolutionary approach to various fields of study, time, and cultural differences. A literature search was conducted using the National Assembly Library, Research Information Sharing Service, National Digital Science Library, DataBase Periodical Information Academic, PubMed, Cumulative Index to Nursing and Allied Health Literature, Excerpta Medica database, and Cochrane.

**Results:**

A total of 28 studies were included, and we categorised the following three attributes: active information seeking, two-way interactive communication, and information utilization/sharing. The antecedents included personal factors, health status, socioeconomic factors, cultural factors, and attitudes toward the Internet while the consequences included increased health interest, health behaviour promotion, and active decision-making.

**Conclusions:**

As e-health literacy in older adults affects their health and quality of life, this study clarifies the concept and provides a conceptual framework for nursing practice and research. Further studies are needed to identify and expand the constantly evolving concept of e-health literacy in older adults.

## Background

Older adults have a higher risk of disease and decline in physical function as they age; moreover, they may experience many acute and chronic problems requiring continuous management by various medical professionals in different environments [1]. In addition, the global life expectancy in 2019 was 73.4 years, and healthy life expectancy was 63.7 years [2]. In this context, older adults likely prioritise a healthy life over a mere extension of their lifespan. Further, many older adults seek health information to maintain their health and treat diseases. However, although health information is provided through various channels, it is difficult for older adults to understand and manage their health using health information without proper guidelines or interventions because of its complexity [3]. Therefore, it is essential to understand the basic literacy related to health information of older adults [4].

Since the 1990s, access to health information has increased due to developments in information and communication technology (ICT) [5]. Moreover, since then, the terms ‘e-health’, ‘ehealth’, and ‘electronic health’ have appeared in literature [6]. In the 2000s, electronic health (e-health) was defined as the use of emerging ICT, especially the Internet, to improve or enable health and health care [6]. Today, e-health has been broadly expanded to include service contents, health care providers, health consumers, and systems [7]. Thus, the need for e-health using ICT has increased [8], and the role of e-health information is becoming more important [9].

The growth in the community-dwelling older adult population and their expectations for patient-centred services have increased the need for the development and use of new information technologies [10]. Older adults can now access health information using the Internet due to the wide availability of smartphones and tablets [11]. While older adults started using the Internet later than younger generations, their usage is increasing rapidly with greater access to computers and the Internet [12]. Furthermore, health information technology use among older adults in the United States of America has increased by about 19%, from 24.8% in 2009 to 43.9% in 2018 [13]. Given that the prevalence of health problems is higher in older adults than in young adults, seeking and utilizing health information over the Internet can be particularly beneficial to them [14]. E-health information can increase access, especially for older people living in isolated rural communities [15]. Older adults can use the Internet to help manage their health, such as making health-related decisions by searching for health information, communicating with medical professionals, seeking health services, and participating in health programs [9].

Early establishment of e-health mostly referred to health service and systems rather than the health of individuals [16]. Since 2010, due to the expansion of electronic health resources to websites, web-based applications, and mobile applications, an imbalance in access to health and medical resources has occurred. Health consumers who have difficulties accessing e-health information showed a marked difference in appropriate self-care and self-management of their conditions [17]. Therefore, older adults should use the Internet to identify health information to manage, maintain, and improve their own health and health care [18].

The term ‘health literacy’ was first used in the United States in 1974 to refer to guidelines for health education among students [19] and refers to an individual’s ability to obtain, process, and understand basic health information and services necessary to make appropriate health decisions [20]. Norman and Skinner [21] proposed an e-health literacy model, defining e-health literacy as the ability to seek, find, understand, and appraise health information from electronic sources. According to this model, e-health literacy includes all aspects of traditional literacy and numeracy, media, computer, information, and science literacy, including traditional health literacy [21]. With the further development of information, the field of e-health has been broadly expanded from service contents, providers, users, and other major systems, and the term ‘digital health’ has recently been widely used [22]. According to the WHO definition, digital health encompasses emerging fields such as big data, genomics, and the use of advanced computing science in artificial intelligence as well as mHealth and e-health. However, e-health literacy is an important indicator of personal health technology utilization [23]. If the level of e-health literacy is low, it is considered that it will be difficult to have digital health literacy. Therefore, in this study, rather than a technical approach, we would like to focus on e-health literacy at the individual level to identify the determinants of the promotion of health management for older adults and to provide a strategy to make good use of e-health resources.

Concept analysis of health literacy was performed in order to clarify its meaning [24], particularly in older adults [25]. Previous studies of e-health literacy were initially conducted in the field of health information technology, such as electronic information records and decision-making systems [26]. Given the progress of electronic devices, studies have been conducted to evaluate health outcomes using mobile devices such as smartphones and tablets [27]. In addition, based on the concept of literacy, a tool to measure the e-health literacy of the youth population was developed in 2006 [21], and a study successfully validated e-health literacy of the elderly using this tool [14].

However, e-health literacy is diverse by race, country, and culture of older adults, and the level of e-health literacy varies across countries, contents, strategies, training tools, and manuals of e-health literacy interventions [28]. Previous studies have not yet been able to present advanced concepts, scope, or standards that reflect the rapidly changing characteristics of e-health literacy, and the concept of e-health literacy of older adults reflecting their characteristics has not been defined. Moreover, as it is used interchangeably with terms such as ‘mHealth literacy’ and ‘digital health literacy’ [7], researchers apply different concepts and definitions [29–34]. This situation also raises questions about the reliability and validity of the study [35]. It is necessary, therefore, to clarify the concept of e-health literacy by identifying the attributes, related concepts, and influencing factors of e-health literacy among older adults.

Clarifying the nature of changes in a dynamic manner through time and context is an important process in the development of useful and meaningful concepts in this field of study [36]. Identifying attributes of e-health literacy in older adults through multidisciplinary comparisons provides evidence for developing interventions for health promotion in the various nursing fields and, in turn, guides the direction of future research. As e-health literacy in older adults is affected by sociocultural and historical factors, such as age, the period of Internet dissemination, and the generalised environment, the concept of e-health literacy should be defined through the evolutionary method proposed by Rodgers [36], which approaches concepts through context and an evolving point of view. E-health resources are constantly evolving and require ongoing adaptation by users [37]. Therefore, this study aims to provide basic data for future research by examining the literature on e-health literacy in older adults based on Rogers’ evolutionary method and identifying its properties, antecedent factors, and results in the literature.

## Methods

### Design

This study is a concept analysis of the attributes, antecedents, and consequences of e-health literacy in older adults using Rodgers’ evolutionary method [36].

## Researchers’ preparation

This study was conducted by researchers with clinical experience with older adults, with gerontological nurse certifications, who have submitted a thesis on older adults, and who have written an article by conducting research related to health literacy. Based on this understanding of older adults, the literature on e-health literacy among older adults was searched and read repeatedly. In this process, we recognised the necessity of a concept analysis of e-health literacy among older adults. Accordingly, researchers attended a doctoral nursing theory development strategy class to acquire knowledge on concept analyses. Further, by reading Rodgers’ [36] literature related to evolutionary theory, we attempted to understand and analyse the philosophical background and analysis methods. In addition, the process was implemented with the advice of experts who had experience in concept analysis as well as those who were educated in nursing theory development.

### Concept analysis process

This study was sequentially analysed using the evolutionary concept analysis method suggested by Rodgers [36]; each step was repeatedly cycled and analysed until the concept became clear. The following steps were followed in the study:

1. Identify the concept of interest and associated expressions (including surrogate terms).
2. Identify and select an appropriate realm (setting and sample) for data collection.
3. Collect relevant data (the attributes and contextual basis of the concept, including interdisciplinary, sociocultural, temporal, antecedent, and consequential occurrences) to identify variations.
4. Analyse data on the above characteristics of the concept.
5. Identify an exemplar of the concept, if appropriate.
6. Identify implications, hypotheses, and ideas for further development of the concept.

### Identifying the concept of interest

The literature was drawn from the time the concept of e-health literacy first appeared until April 2021. For Korean literature, the National Assembly Library, Research Information Sharing Service, National Digital Science Library, and DataBase Periodical Information Academic were used. For English literature, PubMed, the Cumulative Index to Nursing and Allied Health Literature, Excerpta Medica database, and Cochrane search engine were used. ‘E-health literacy’ and ‘electronic health literacy’ were searched as keywords, and ‘computer/internet/mobile/online AND health literacy’ were included to cover a wide range of literacy on devices. In addition, ‘older adults/elderly/senior/baby boomer/retiree/pensioner’ were added to limit search results as a final outcome to identify the attributes, antecedents, and consequences of e-health literacy in older adults. However, the age range of older adults was not limited because it differed from study to study in extant literature.

### Choosing the setting and sample

To analyse the concept of e-health literacy, quantitative and qualitative research was extensively considered, focusing on empirical studies. The inclusion and exclusion criteria were as follows.

1. Inclusion criteria
  - Studies published from the time the concept first appeared until April 2021
  - Studies that further the understanding of the concept and attributes of e-health literacy among older adults
2. Exclusion criteria
  - Studies not targeting older adults
  - Studies on older adults with certain diseases
  - Studies not related to e-health literacy
  - Literature not published in either English or Korean
  - Grey literature including books, materials, and pilot studies
  - Literature for which no abstract or original text could be found
  - Not suitable for the main research purpose

### Data analysis

We organised selected literature using a matrix [38]. This structured analysing matrix was used to distinguish research fields, research purposes, research designs, inclusion of the concepts, and keywords. Moreover, to determine attributes of e-health literacy, major questions including ‘What is e-health literacy for older adults?’ and ‘What are the characteristics and features of e-health literacy of older adults?’ were repeatedly discussed during regular meet-ups with researchers. In the matrix, articles with vague description in attributes were set aside, and some studies in other professional fields were also reappraised. Then, all antecedents, attributes, and consequences were integrated with original results, and whether it was situation, event, or phenomenon was confirmed before and after the concept. In this process, analysed the attributes of the concept and the evidence of context strictly by asking opinions of other professionals about the categories of antecedents, attributes, and consequences. To derive research results inductively, we attempted to find an adequate exemplar in qualitative study to identify the attributes applicable in reality.

## Results

### Outcomes of searched literature

**Fig. 1:**
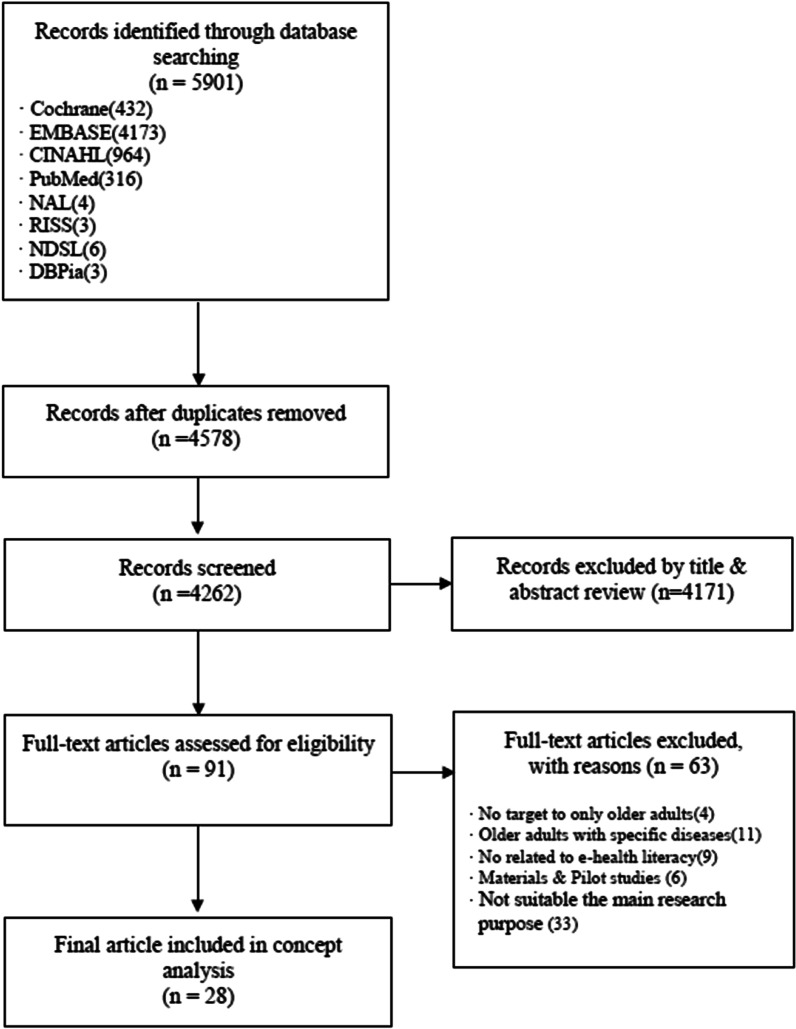
Literature search flow

The researchers (SJ, YS, and EC) extracted 5585 primary studies from the searched literature; 16 were in Korean, and 5569 were in English. Excluding 1323 duplicate studies, 91 studies were derived by screening the titles and abstracts according to the inclusion and exclusion criteria. After reading through the full text of 91 studies, discussions among researchers resulted in the exclusion of 63 studies based on the criteria above. Finally, 28 studies, consisting of 3 Korean and 25 English studies, were analysed in this study (Fig. 1).

### Definition of e-health literacy

To understand the attributes of e-health literacy in older adults, we confirmed the definition of the term used in each study and identified how the term changed over time (Table 1). Early studies’ descriptions of the e-health literacy of older people were similar to the existing definition of health literacy, which includes the abilities to search, understand, and utilise health-related information. However, newer studies gradually reflected the development of information technologies and devices that affected older adults’ ability to respond to health-related information. In addition, some definitions included the characteristics of older adults and the manner in which they identify what they need from various media and reproduce or share such information rather than merely evaluating it. However, most recent studies have used the definition of Norman and Skinner [21], either by translating or quoting a part of the definition.

**Table 1:** The definitions of e-health literacy in older adults

Concept	Citation	Year	Definition	Field
Internet health information	[39]	2005	Older adults would use the Internet to gather health care information and actively seek information that could directly affect their own treatment and care	Health and communication
Internet health information	[40]	2005	Access to Internet as well as the skills necessary to find, retrieve, and evaluate information	Medical internet
Internet Health information literacy	[41]	2013	The abilities to recognize a need for health information, to identify and use likely information sources, and to evaluate, understand, and use the information in order to make good health decisions	Library and information
e-health literacy	[23]	2013	Ability to access information and use the information to support self-management of a health concern	Nutrition
Internet health information	[33]	2013	The ability to enable older people to use the Internet to feel more knowledge and take steps to improve their health	Medical informatics
On-line health seeking activity	[15]	2018	On-line information can be a gateway to meet the healthcare needs of a growing older population, particularly as access to health professionals and health information has been identified as problematic for some older people and this can be improved, particularly for those living in isolated rural communities	Nursing
e-health literacy	[42]	2019	Ability to seek, understand, and evaluate health information desired on the Internet, and to apply online health information to health problems and solve them	Nursing
e-health literacy	[43]	2019	The ability to find, understand, and appraise electronic information on the Internet by self-determining what information an individual need	Nursing
e-health literacy	[44]	2020	The ability to seek, find, understand, and appraise health information from electronic sources and apply the knowledge gained to addressing or solving a health problem	Gerontology
e-health literacy	[17]	2020	The ability to seek, find, understand, and appraise health information on the Internet, as well as the ability to apply and transmit the knowledge gained to deal with and solve health problems	Nursing

### Attributes

**Fig. 2:**
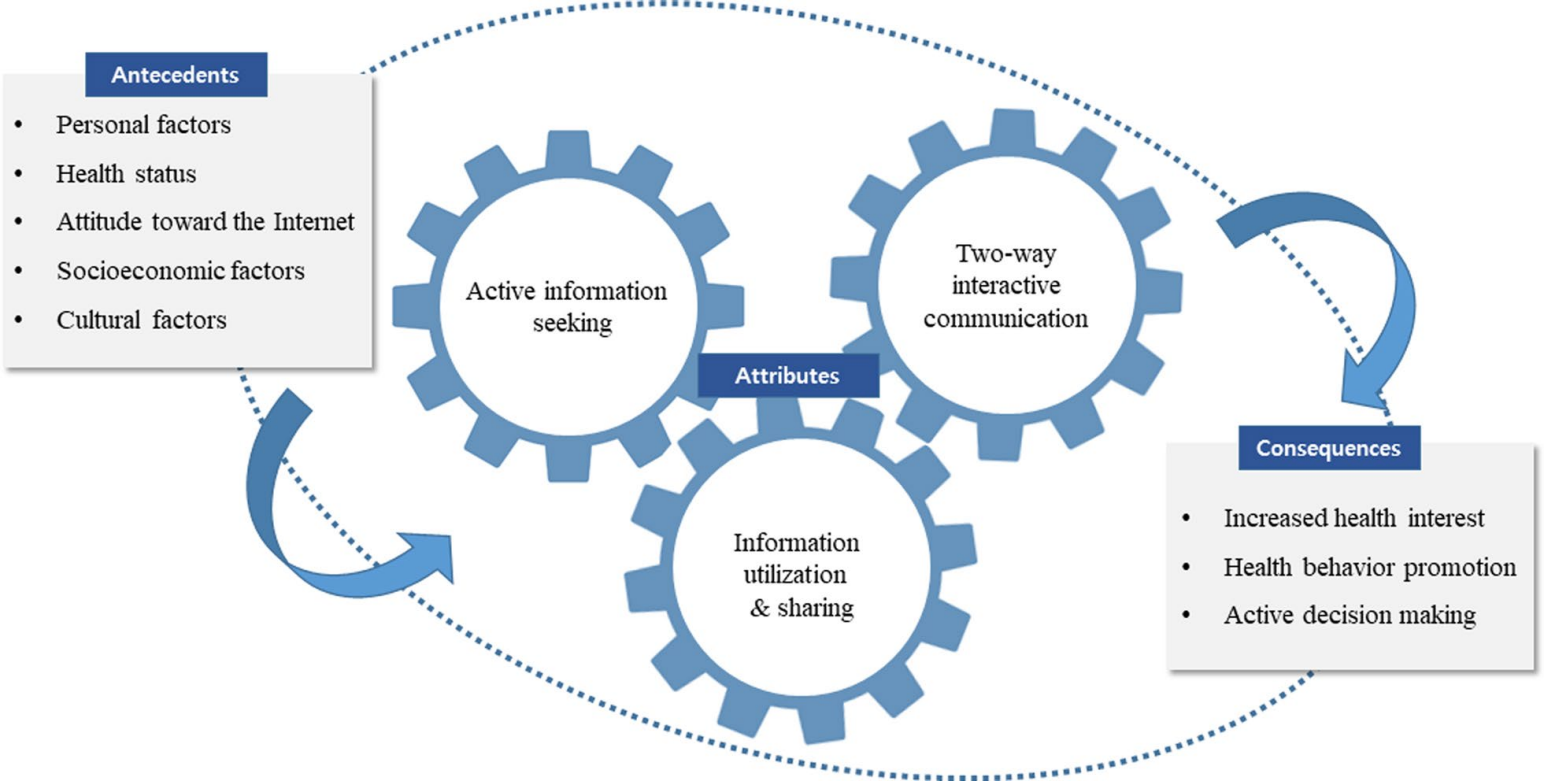
Conceptual framework of e-Health literacy in older adults

Defining the attributes of a concept can help identify a real definition, which is necessary to understand the concept’s characteristics [23]. Moreover, defining attributes is fundamental to concept analysis as it provides the broadest insight into the concept [45]. In this study, we identified the attributes of the concept through the question, ‘What are the consistent characteristics of e-health literacy in older adults?’ The identified attributes of e-health literacy in older adults are active information seeking, two-way interactive communication, and information utilization and sharing (Fig. 2, Table 2).

**Table 2:** Antecedents, attributes, consequences from literature

Dimensions	Sub-dimensions	Findings from literature
Antecedents	Personal factors	Age [13, 17, 29, 31, 33, 34, 41, 44, 46]
		Gender [13, 29, 46, 47]
		Health literacy level [15, 44]
		Prior experience with Internet use [29–31, 41, 48]
		Frequency of internet use [43]
		Prior experience with internet instruction [29, 41]
		Lack of computer skills [15, 41]
		Difficulties to access the internet [15, 29, 30]
	Health status	Vision/Hearing [30, 33, 49, 50]
		Individual health status [41, 43]
		Cognitive/language impairment [30, 49, 50]
	Attitude toward online	Computer anxiety [33, 39, 41, 44, 50]
		Computer self-efficacy [31, 39, 41, 50]
		Computer confidence [41]
		Types of preferred sources [31]
	Social-economic factors	Income [29, 30, 44, 46, 47]
		Education [17, 29, 30, 34, 41, 46, 47, 51, 52]
		Marriage [44, 46]
		Social support [29, 53]
		Number of e-devise [44]
		Need for assistance [30]
	Cultural factors	Perceived usefulness and Zon e-health information [15, 23, 29, 30, 40, 43, 48]
		Perceived health status [13, 17, 34, 54]
		Historical background [29]
Attributes	Active information seeking	Information-seeking needs/recognition the importance of health promotion [23]
		Benefit to well-being [55]
		Seeking Internet health information [17, 53]
		Seeking information in professional website and personal blogs [13]
	Two-way interactive communication	Communication with health care providers [13, 56]
		Real-time interactive communication/no delay with need for speed [32]
		Searching for information to understand what doctors say/information to develop questions without hesitant [33, 39–41]
		Control over online experience/management of information needs [32]
		Bidirectional flow of information and active role in their health care [15, 57]
	Information utilization and sharing	Adaption of dynamic nature of the internet [23]
		Information utilization/availability of a wealth of information [41]
		Acquired familiarity and accessibility [30]
		Application of online knowledge to solve health problem [17]
		Self-belief in an ability to evaluate online health content [55]
		Opportunity to help older adults [44]
		Constantly updated and engaged interactively/delivering self-management and health promotion information [47]
		Scheduled medical appointment on the internet [56]
		Online health information changed continuously and ranged variously [13]
Consequences	Increased health interest	Patient knowledge and expectations regarding health care [29]
		Enhanced knowledge [50]
		Medical equality and inequality [43]
		Bolstered confidence [31, 41, 50]
		Expansion of health interest and expectancy level of health information [17]
	Health behavior promotion	Coping with the stressful situation [58]
		Promoted patient self-management [15]
		Medication usage/physical condition [46]
		Lifestyle changes [33]
		Chronic disease management [31]
		Capacity to engage in health protective behavior [50]
		Prevention diseases/Health maintenance/Health promotion/Improvement of health care quality and outcome [56]
		Overall quality of life [46, 50]
	Active decision making	Understanding/monitoring/complement to health providers’ decisions to choose [58]
		Enhanced communication with health professionals/decisions making of one’s health and self-diagnosis [15]
		Demanding alternative treatments/active participation [41]
		Active decision-making behavior in doctor visit and communication [17]

#### Active information seeking

Older adults recognise the importance of health promotion, along with the need to identify health information [23]. They actively seek information using electronic media when they recognise its benefits for their wellbeing [50]. In other words, when they need the health information, they also identify how to obtain and actively seek it. Older adults search for information online to solve health-related questions from various sources, ranging from professional websites to personal blogs [13, 17, 53].

#### Two-way interactive communication

Health-related information obtained through the Internet allows for real-time interactive communication using electronic media, which facilitates the understanding of various information [32]. This includes searching for information to understand what doctors say, formulating questions for medical professionals without hesitation, and communicating with health care providers [13, 33, 39–41, 56, 58]. In other words, older adults play an active role in their health management, using two-way interaction to understand the information they receive [15, 57]. Moreover, they check the necessary health needs in real time to self-manage the discovered information [32].

#### Information utilization/sharing

Older adults evaluate the information that they receive based on their understanding [50] and apply online knowledge to solve their own health problems [17]. In addition, information is constantly updated and modified after the searched knowledge is conveyed to online information providers or others seeking information [13, 31]. This circulates in the desirable direction of participation as older adults share and reproduce this health information among other older adults online [13, 44] rather than passively accepting and using it privately.

### Antecedents

Antecedents are events that occur prior to the concept [36]. They were classified into personal factors, health status, attitudes toward the Internet, and socioeconomic and cultural factors by reading the literature in view of the question, ‘What are the events that occur prior to e-health literacy?’ (Fig. 2, Table 2).

#### Personal factors

Prior literature confirms the effect of personal characteristics of older adults on e-health literacy. We found that the younger the age [41, 44] and the higher the level of health literacy were [15, 41, 44], the more positive was the effect on e-health literacy. In addition, e-health literacy was high in those who personally had prior experience with Internet use [30, 31, 41, 48] or Internet instruction [29, 41]. However, those who lacked computer skills [15, 41] and experienced difficulty in accessing the Internet for health knowledge [15, 29, 48] had low e-health literacy.

#### Health status

As aging progresses, older adults may experience loss of vision, hearing, and mobility [15, 30]; further, their cognitive function and language ability may decline [30, 55]. Thus, the individual health status of older adults [13, 17, 34] is a key factor influencing e-health literacy.

#### Attitudes toward the internet

Attitudes toward the Internet influenced e-health literacy in older adults. The absence of computer anxiety [23, 39, 41, 44, 50], higher computer confidence [44], and computer self-efficacy [31, 39, 41, 50] enhanced e-health literacy among older adults.

#### Socioeconomic factors

Studies reveal that higher income [30, 46] resulted in having a higher number of devices [44]; further, the higher the education level was [32, 52], the higher was the level of e-health literacy. Older adults with high incomes were more likely to have many electronic devices. Moreover, the higher the educational attainment among older adults, the more likely they were to have basic literacy, thus requiring limited assistance in using computers. In addition, if older adults were married [44, 52] and had social support [53], they were able to receive help with computer use [30], which resulted in high e-health literacy.

#### Cultural factors

Perceived usefulness of and trust in e-health information [15, 23, 29, 30, 40, 43] improved e-health literacy. Moreover, older adults who had experienced historical events such as war, baby boom, and economic development [29] confirmed that these cultural factors affect the level of education and, consequently, e-health literacy.

### Consequences

Consequences of the concept refer to events or phenomena after the concept occurs [36]. We identified the consequences through the question, ‘What is the consequential occurrence of e-health literacy in older adults?’ In doing so, we derived the consequences of increased health interest, health behaviour promotion, and active decision-making (Fig. 2, Table 2).

#### Increased health interest

E-health literacy in older adults increases interest in health and enhances knowledge and expectations of health care [29, 50]. It induces health information pursuit, confidence in Internet health information [50, 51], and a positive attitude [17]. These circumstances may lead to an increased health interest [43].

#### Health behaviour promotion

The ability to apply the acquired knowledge to address and solve health-related problems can be increased by e-health literacy among older adults, which can ultimately improve their health status. These behaviours include managing chronic disease [31], changing lifestyle [33], and improving their self-management abilities [15]. However, older adults with lower e-health literacy not only lack information but also experience medical inequalities in relation to the availability of health resources [52]. Moreover, another study found that enhancing coping ability for stressful situations increases older adults’ understanding of medication usage [58], and identifying behaviours for disease prevention and health maintenance [43, 50] could ultimately improve their overall quality of life [46, 50].

#### Active decision-making

The process of accurately recognizing one’s health status by using electronic health resources allows older adults to actively make treatment-related decisions. E-health literacy helps older adults in determining which health care provider to choose [58] while enabling them to understand and complement the staff’s decisions [58], thereby strengthening the communication between service provider and user [15]. Moreover, it encourages older adults to actively participate in the treatment process [17, 46], thus inducing positive changes in the treatment process [46].

### Related concepts and surrogate terms

Identifying a variety of related concepts or surrogate terms is crucial to the interactive development of concepts [36]. Surrogate terms are means of expressing a concept other than the word or expression selected by the researcher to focus the study [36]. Surrogate terms must be identified to some extent before beginning formal analysis [36]. Surrogate terms checked before the official analysis were ‘mHealth literacy’ and ‘digital health literacy’, and it was confirmed that they were used interchangeably with ‘e-health literacy’. E-health literacy is a key attribute of these concepts, and, in this study, only the literature focusing on e-health literacy was extracted. We identified such terms in each paper. Especially in South Korea, the phrases ‘*ability to understand Internet health information*’ and ‘*Internet health information literacy*’ have been frequently used as surrogate terms.

In this study, Related concepts that contain some important properties of the concept or are similar but not identical to the concept were described as well as how they are connected to the concept network surrounding the concept [36]. The attributes of concepts were different from those of e-health literacy or contained only some parts of the attributes. For example, older adults’ ‘*access to health information usability and accessibility*’ [34] or ‘*digital access to health information*’ [29, 31] was used to describe the ability to access information through the Internet. In addition, ‘*online health information seeking*’ [31, 48, 55, 57] was conceptually described as the process of searching for information. Moreover, the concepts for older adults’ acceptance of information were developed and used. They included ‘*acceptance and use of health information technology*’ [13, 30, 31] and ‘*Internet-based interactivity information*’ [33]. The concept of ‘*computer literacy*’ [32] appeared with the development of electronic devices. Therefore, it is important to clearly distinguish attributes in each concept as they influence the conceptual development of e-health literacy.

### Model case

According to Rogers [36], this analysis method was performed inductively; thus, in order to verify the concept of the study, it was necessary to confirm the actual example and apply the attributes, antecedents, and consequences to present them. Therefore, in this study, the use of Internet media in situations of need in older adults was presented as a model case [58]:

When my wife had ovarian cancer, I became relatively knowledgeable about it. When we went to a surgeon, I asked the surgeon some questions and determined very quickly that I knew more about it than he did, and we just walked out. I went to the web, and I looked it up.... I found the list of names of the top-rated oncologists in the area, and the same way with the top-rated medical centres.... As a result, we talked with the top doctors at one of the top centres in this area. When I was growing up, you went to the doctor, did what he said, and took the medicine he gave you, and just believe; don’t ask questions, just do it.... Things are done a little bit differently these days. In the past, doctors were accepted on faith, but now, you could have a dialog with a doctor.

## Discussion

This study aimed to analyse e-health literacy using an evolutionary approach to identify the definition of e-health literacy while demonstrating the contextual flow of antecedents and consequences. The results showed that e-health literacy includes attributes such as active information seeking, two-way interactive communication, and information utilization and sharing. Characteristics related to personal factors, health status, attitudes toward the Internet, socioeconomic factors, and cultural factors of older adults were identified as antecedents of e-health literacy. As a result, we found that e-health literacy enhanced health care interest, promoted health behaviour, and induced active decision-making in older adults, which ultimately improved their quality of life.

### Principal results

First, from personal factors, the e-health literacy of young adults increased with age [41, 44], but the e-health literacy of older adults decreased with age [59]. As noted in the previous literature, this is likely because older adults are more likely to experience vision and hearing loss [15, 49], decline in cognitive ability [15], and decreased individual health status [41, 43].

In addition, previous studies noted that higher levels of health literacy in older adults result in greater acquisition of e-health literacy [30, 46], which is a concept that includes the various abilities required to control health-related problems in daily life [60]. This suggests that promoting health status allows older adults to explore and utilise health-related information through electronic devices. Older adults’ attitudes toward the Internet are also an important factor in e-health literacy; these include computer anxiety, computer self-efficiency, computer confidence, and other types of preferred sources. Further, prior experience with Internet use and instruction, increased frequency of Internet use, and familiarity with online media use can reduce difficulties related to computer skills and internet access [15]. It is thus necessary for researchers to study and policymakers to develop interventions to increase older adults’ confidence and self-efficiency in electronic media use, reduce fear of Internet and computer use, and increase accessibility.

Socioeconomic factors suggest that, as in previous studies, older adults with higher social status, educational backgrounds [32, 53], economic conditions, and marriage status and social support [46] have relatively high levels of e-health literacy. As e-health literacy is influenced by educational attainment [55], affecting older adults’ cognitive ability to understand, assess, and apply their knowledge, it is important to approach individual older adults based on their education levels [61]. In addition, those with higher incomes are more likely to own or have access to electronic devices, while, on the other hand, older adults with lower incomes have less opportunity to access electronic health information [30]. The presence of marriage and social support in older adults’ lives also affects e-health literacy as family and other social relationships can help them use electronic media to search for information on their own [44]. This suggests that older adults who live alone or isolated may be vulnerable in their ability to understand and utilise e-health resources due to the absence of support around them, indicating that public healthcare approaches should be applied to remove this inequity.

Cultural factors indicate that older adults perceive their health conditions and start to seek useful e-health information, which is influenced by environmental factors, such as the region, country, and culture in which the individuals live [61]. In particular, as older adults have many restrictions regarding movement around their residential areas, cultural factors can play an important role in recognizing online health information [29]. In addition, when applying the concept, historical background must be considered as it can also affect e-health literacy. For example, people born in the 1950s, who were not educated due to the Korean War, had relatively low literacy levels, which had a negative impact on their current levels of e-health literacy as older adults [29].

From the attributes of e-health literacy in this study, active information seeking refers to the act of exploring using electronic media for health information. It is based on the need for and perception of finding health information that would be beneficial to older adults’ health [55].

In modern society, the growing interest in health and active health care of older adults has evolved into a form of interaction with experts or others through various media. This has moved away from a unilateral process of information acceptance and affords an opportunity for older adults to choose what they utilise and share with others on their own [9]. After all, among physical and technical limitations of older adults, only literacy levels were assessed in early studies; real-time information exchange has given online communication an important role in recent studies [15]. Older adults may direct their queries to health professionals when they are unable to understand health information on the Internet through the Internet community and mobile applications. They actively exchange their knowledge and experiences with adults from other regions, genders, and age groups without space–time constraints. In addition, instead of meeting health professionals to obtain limited information, online real-time hospital appointments, consultations, and medical results confirmation by themselves have become available [32].

In the process of accessing the dynamic nature of the Internet, older adults can have abundant information to apply to health-related problems. Additionally, from that experience, they gain self-belief in evaluating online health content [50]. Reproducing information by continuously sharing and updating information can have a positive impact on other older adults as well as promoting their own health [44]. However, this diverse and continuous process of e-health literacy may vary depending on the different Internet technology environments in each country [28]. While older adults need to acquire the ability to select accurate and appropriate information from the abundant information that is indiscriminately shared online, standards for information can also vary depending on attitudes or social conventions.

As Internet use continues to increase, it is important to approach and apply intervention through the concepts to improve e-health literacy in older adults [18]. As some individuals have difficulty accessing and utilising health information due to personal capacities, training on new skills should be provided at the individual level for better health outcomes [62]. In addition, identifying the factors that promote and inhibit e-health literacy in older adults and implementing effective interventions to improve their abilities will contribute to diminishing the gap in health literacy [54].

This study indicated that older adults, who previously solved their health problems passively and had insufficient opportunities to gain health information, now maintain a more active life by interacting and sharing information with others in the common context of health promotion. In addition, older adults with high e-health literacy have increased interest in health and are aware of how to maintain their health or prevent disease through health-related information on the Internet. Therefore, it is expected that e-health literacy of older adults will be improved if nursing care is provided using antecedents identified in this study. We propose to develop tools and intervention programs for older adults that reflect the attributes of e-health literacy identified in this study. Furthermore, practical interventions such as the development of educational programs based on multidisciplinary convergence are required. This study is significant in that it provides basic data necessary for providing nursing care to increase the e-health literacy of older adults.

### Limitations

The limitations of this study are as follows. First, the age boundaries of older adults are unclear as each study defined age groups according to the population’s sociocultural context. Therefore, one should be cautious while generalizing the findings of this study. Second, our study was aimed at older adults in general; follow-up research is needed to identify the attributes of older adults with certain diseases as well as the changes in the information available on the Internet. Finally, as this study analysed concepts according to the contextual and temporal trends that have occurred so far, it is necessary to apply the evolutionary concept analysis method to continuously identify and extend the changing attributes of e-health literacy in the future.

## Conclusions

This study is a concept analysis that confirms the meaning and attributes of e-health literacy among older adults using the evolutionary method to derive a conceptual definition of e-health literacy for older adults. The main attributes identified in this study were active information seeking, two-way interactive communication, and information utilization and sharing; moreover, we confirmed that these attributes were organically related. Therefore, e-health literacy of older adults can be defined as actively searching for necessary health information using electronic media, exchanging real-time information, and promoting one’s own health by utilizing and sharing it. Therefore, based on these research results regarding e-health literacy, follow-up studies on measurement development and additional research are needed to reveal changes in concepts that evolve with the development of electronic resources in the future.

## Data Availability

Not applicable.

## Abbreviations

ICT: Information and communication technology
E-health: Electronic health
